# Genomic Signatures of Positive Selection in Human Populations of the *OXT*, *OXTR*, *AVP*, *AVPR1A* and *AVR1B* Gene Variants Related to the Regulation of Psychoemotional Response

**DOI:** 10.3390/genes14112053

**Published:** 2023-11-08

**Authors:** Siroj Yu. Bakoev, Anna V. Korobeinikova, Arina I. Mishina, Shuanat Sh. Kabieva, Sergey I. Mitrofanov, Alexey A. Ivashechkin, Alexsandra I. Akinshina, Ekaterina A. Snigir, Sergey M. Yudin, Vladimir S. Yudin, Lyubov V. Getmantseva, Elmira A. Anderzhanova

**Affiliations:** Federal State Budgetary Institution “Centre for Strategic Planning and Management of Biomedical Health Risks” of the Federal Medical Biological Agency (Centre for Strategic Planning of FMBA of Russia), Pogodinskaya Street, 10, Bld. 1, 119121 Moscow, Russia; akorobeinikova@cspfmba.ru (A.V.K.); amishina@cspfmba.ru (A.I.M.); shkabieva@cspfmba.ru (S.S.K.); mitrofanov@cspfmba.ru (S.I.M.); aivashechkin@cspfmba.ru (A.A.I.); akinshina@cspfmba.ru (A.I.A.); esnigir@cspfmba.ru (E.A.S.); yudin@cspmz.ru (S.M.Y.); vyudin@cspfmba.ru (V.S.Y.); lgetmantseva@cspfmba.ru (L.V.G.); eanderzhanova@cspfmba.ru (E.A.A.)

**Keywords:** genomic signatures, positive selection, adaptation, stress, *OXT*, *OXTR*, *AVP*, *AVPR1A*, *AVPR1B*

## Abstract

The neurobiological systems of maintenance and control of behavioral responses result from natural selection. We have analyzed the selection signatures for single nucleotide variants (SNV) of the genes of oxytocin (*OXT*, *OXTR*) and vasopressin (*AVP*, *AVPR1A*, *AVPR1B*) systems, which are associated with the regulation of social and emotional behavior in distinct populations. The analysis was performed using original WGS (whole genome sequencing) data on Eastern Slavs (SlEast), as well as publicly available data from the 1000 Genomes Project on GBR, FIN, IBR, PUR, BEB, CHB, and ACB populations (the latter were taken as reference). To identify selection signatures, we rated the integrated haplotype scores (iHS), the numbers of segregating sites by length (nSl), and the integrated haplotype homozygosity pooled (iHH12) measures; the fixation index Fst was implemented to assess genetic differentiation between populations. We revealed that the strongest genetic differentiation of populations was found with respect to the *AVPR1B* gene, with the greatest differentiation observed in GRB (Fst = 0.316) and CHB (Fst = 0.325) in comparison to ACB. Also, high Fst values were found for SNVs of the *AVPR1B* gene rs28499431, rs33940624, rs28477649, rs3883899, and rs28452187 in most of the populations. Selection signatures have also been identified in the *AVP*, *AVPR1A*, *OXT*, and *OXTR* genes. Our analysis shows that the *OXT*, *OXTR*, *AVP*, *AVPR1A*, and *AVPR1B* genes were subject to positive selection in a population-specific process, which was likely contributing to the diversity of adaptive emotional response types and social function realizations.

## 1. Introduction

Identification of positive selection genomic signals is a traditional population genetics approach based on the analysis of results of whole genome sequencing (WGS) from the perspective of comparison between species and populations. The signals indicate genetic variants corresponding to different adaptation models and, therefore, designate loci of phenotypic variability, thus appearing as objects of particular interest when mapping complex traits [1,2,3].

The driving forces and direction of selection during the evolution process are not always clear. Nevertheless, the development and human populations’ growth upon the settlement of *Homo sapiens* in various landscape and climatic zones [4] came to a realization through the accumulation of the variants that are most suitable for new environments and contributing to the reinforcement of specific patterns of cooperative behavior and offspring care [5]. Many of the selected variants can affect complex endophenotypes (characteristic sets of behavioral signs with a strong genetic component), which represent constellations of normal or altered emotional, social, and cognitive functions [6] and which, among others, can be determined by gene pleiotropy, hysteresis, and other phenomena that define the complex trait’s variability.

At present, there is growing interest in understanding the genetic basis for emotional and sociocognitive functions in s [7,8,9,10]. With regard to social and emotional behavior, the neuropeptides oxytocin (OXT), vasopressin (AVP) and their receptors (AVPR1A, AVPR1B, and OXTR) gain particular attention [11,12] because the role of OXT and AVP in the mechanisms of establishing and maintaining social interaction, social memory, empathy, prosociality, and anxiety is well recognized [11]. Mechanisms of psychoemotional adaptation are gaining attention due to the standing problem of acute and chronic stress and the aging of the general population. Therefore, both genetic factors and molecular-biological mechanisms of stress resilience and susceptibility to environmental changes evoke persistent interest. A review of the molecular mechanism of oxytocin and vasopressin action can be found in [13,14].

It is of note that the *OXT* and *AVP* genes show almost no variability in their sequences, while the genes encoding their receptors are characterized by a high degree of variability (the high number of SNVs associated with phenotypic peculiarities). Therefore, the study on variability of the *AVPR1A*, *AVPR1B*, and *OXTR* genes makes a great contribution to understanding the genetic architecture of behavioral traits associated with social behavior and emotions [15]. It has already been shown that genetic variants in the human *AVPR1A*, *AVPR1B,* and *OXTR* genes are associated with the diversity of perception of social signals (empathy, emotional intelligence, eye contact, face “reading,” autism), prosociality (trust, altruism, generosity, loyalty), social tolerance (aggressiveness, competition), and anxiety [7,8,16,17] that contributes to the variability of personality types appearing in human population. In turn, evaluation of the positively selected variants is of particular interest because it allows one to identify functionally significant SNVs contributing to the formation of psychopathological phenotypes.

The signaling mediated by oxytocin and vasopressin is essential not only in the regulation of physiological functions, among which labor in females and reabsorption of water in the kidney, respectively, are most prominent. Polymorphisms in genes for oxytocin and vasopressin and their specific receptors are associated with diversity in traits related to the behavioral functions of positive and negative valence and general arousal. They are significant in setting the cumulative risk for autism spectrum disorders (ASD), schizophrenia, stress susceptibility and panic disorder, major depression, and aggression [18,19,20,21,22,23,24]. The high biomedical significance of both signaling systems mediating psychoemotional functions and social interactions supports a persistent interest in the genetics of these two systems.

Identification of positive selection genomic signals in distinct populations would be helpful in revealing genetic mechanisms of behavioral traits. The study was based on the assumption that diversity in the climate/geographical zones is a factor of difference in the scenarios of positive gene selection during *Homo sapiens* migration. Therefore, the genetic diversity within the genes of the oxytocin and vasopressin systems across different human populations was our primary interest. In this study, we identified selection signatures in the *OXT*, *OXTR*, *AVP*, *AVPR1A*, and *AVPR1B* genes in different human populations with population genetic models. 

## 2. Materials and Methods

### 2.1. Characteristics of Sample

An original sample of 50 individuals from the East Slavic subethnic group (SlEast) was stratified based on the criteria, which were set upon results of the principal component analysis (PCA) of the 1000 Genomes consortium data (Figure S1) [25]. In addition, we performed the cluster analysis by admixture to show the ancestry proportion of SIEast versus the 1000 Genome samples (Figure S2).

WGS data for Afro-Caribbeans in Barbados (ACB), British from England and Scotland (GBR), Finns in Finland (FIN), Iberian population in Spain (IBR), Puerto Ricans in Puerto Rico (PUR), Bengalis in Bangladesh (BEB), and Han Chinese in Beijing (CHB) (50 individuals in each ethnic group) were from the 1000 Genomes Consortium public database. Fifty individuals from each ethnic group were selected in a random order from the general pool of data.

### 2.2. DNA Isolation, Preparation of Sequencing Libraries and WGS of Whole Blood Samples

Isolation of genomic DNA (gDNA) from whole blood samples was performed manually using a DNA Blood Mini Kit (Qiagen, Hilden, Germany) in accordance with the manufacturer’s protocol. The yield and purity of the isolated gDNA were manually determined using Qubit 4.0 fluorimeter (Thermo Fisher Scientific, Waltham, MA, USA) and NanoDropTM One C Microvolume UV-Vis (Thermo Fisher Scientific, MA, Waltham, USA), respectively. Only gDNA samples with absorbance ratios A260/280 of 1.7–1.9 and A230/260 of 1.8–2.2, in accordance, were selected for further analysis.

The amount of 150–500 ng of gDNA was used to prepare Next-Generation Sequencing (NGS) libraries. Libraries were prepared using the Illumina DNA Prep kit (Illumina, Inc., San Diego, CA, USA) according to the manufacturer’s recommendations using the Tecan Freedom EVO robotic station (Tecan, Männedorf, Switzerland). The gDNA concentrations in library samples were measured using the Infinite F Nano Plus tablet reader (Tecan, Männedorf, Switzerland). The size of the resulting libraries was determined with an Agilent D1000 reagent kit using an Agilent 4200 TapeStation (Agilent Technologies, Inc., Santa Clara, CA, USA). Pooling was performed automatically using a Tecan Freedom EVO robotic station (Tecan, Männedorf, Switzerland). Each of the 50 samples of the library pool was diluted to the final gDNA concentration of 1.5 nM prior to sequencing. Pool quality control was performed with an Agilent High Sensitivity D1000 Screen Tape reagent kit using an Agilent 4200 TapeStation (Agilent Technologies, Inc., Santa Clara, CA, USA).

WGS was performed on an Illumina NovaSeq 6000 (Illumina, Inc., San Diego, CA, USA) with an S4 reagent kit (Illumina, Inc., San Diego, CA, USA) upon 300 cycles with 2 × 150 bp paired-end reads with at least 30× average depth of coverage (>350 million reads). The Illumina Dragen Bio-IT platform (Illumina, San Diego, CA, USA) was used to align reads to the reference genome (GRCh38).

### 2.3. Population Genetic Analysis

The choice of analytical approaches was based on their specificity to spot various characteristics of the selection signal. In general, selection signals are subdivided into two types: “hard” selection, in which de novo mutations are fixed and the frequency of specific haplotypes is significantly increased in a population, therefore eliminating genetic diversity in the vicinity of the adaptive locus [26]; and “soft” selection, when selection acts toward the previously neutral alleles or allele frequency increases, resulting in a higher frequency of a specific set of haplotypes [1,25]. Respectively, we used the following statistics to look for regions of recent and/or ongoing positive selection. (1) iHS (integrated haplotype score) identified loci in which a strong selection led to the fixation of new alleles and to the decrease in haplotype homozygosity (alleles that may be on their way to fixation or become a balanced polymorphism) [1]. (2) nSl (number of segregating sites by length), which generally serves the same purposes as iHS, allowing one to detect hard and soft selective sweeps, appears more stable, especially with regard to the difference in the rate of recombination and the influence of population factors such as population splits, population bottlenecks (when a sharp decrease in the size of the population results in a significant drop in the genetic variability), and population growth without requiring a genetic map [8]. (3) iHH12 (integrated haplotype homozygosity pooled), which was developed based on H12 statistics [27], is able to scan soft selective sweeps by adapting H12 into an integrated haplotype homozygosity structure [28]. Finally, (4) the fixation index Fst was used to assess the genetic differentiation of populations [4]. Because a local adaptation of a population can appear even at conditions of low genomic differentiation, a comparison of genetic variability between two populations increases the power of detection for positive selection, in contrast to what can be revealed at the level of a single population.

Quality control, cohort pooling, and removal of duplicate SNVs were performed in accordance [29]. Data for all 400 (eight populations, each represented by 50 individuals) samples were combined using the *plink1.9* program [30]. *bcftools* software [31] was used to remove duplicate SNVs with identical positions. Duplicates were removed to provide effective processing on the next steps of analysis. After variant calling, variants where more than 5% or 10% of the genotype calls were not available, were removed with *geno0.05* and *mind0.1* tools, respectively. Hard-filtering of rare minor alleles was performed with *maf0.05*. Within-group data pooling was performed after removing multiallelic SNVs and correcting allele flipping. All data were normalized to the GRCh38 reference. The start and end positions for the *OXT*, *OXTR*, *AVP*, *AVPR1A*, and *AVPR1B* gene regions (GRCh 38 assembly) were obtained from the NCBI database (accessed on 15 August 2022) [32].

Genetic variability was assessed in every population to weight intrapopulation variability. The iHS, nSl, and iHH12 approaches for the detection of selection signals were implemented using the *selscan* program [33]. SNVs with a minor allele frequency (MAF) > 5% were kept for analysis, with each SNV sequentially treated as a major SNV. Non-standardized scores were normalized using the “norm” script of the *selscan* program. Windows with crit = 1 values were considered as candidate regions for selection.

We have compared a number of different populations with the ACB population aiming at the evaluation of changes in genomes, which were accompanying the migration of *Homo sapiens* from Africa and its accustoming to new regions of settling. The genetic differentiation of the ACB and SlEast, ACB and GBR, A and CB and FIN, ACB and IBR, ACB and PUR, ACB and BEB, and ACB and CHB populations for the *OXT*, *AVP*, *AVPR1A*, *AVPR1B*, *OXTR* genes was calculated based on the pairwise Fst method proposed by Weir & Cockerham, which was implemented in the *VCFTools* program [34]. A difference was considered significant at Fst ≥ 0.3, which corresponds to the quantile level of 0.99 [35].

## 3. Results

### 3.1. Overall Estimation of Population Differentiation Based on the Analysis of Genes of Oxytocin and Vasopressin Systems

The greatest genetic population differentiation was observed with respect to the *AVPR1B* gene. The average Fst value for all analyzed populations was of 0.277 with the differentiation of GRB to ACB and CHB to ACB of Fst = 0.316 and Fst = 0.325, respectively. Selection signatures have also been identified in the *AVP*, *AVPR1A*, *OXT*, and *OXTR* genes. The Fst values of population differentiation averaged among all variants of the *OXT*, *OXTR*, *AVP*, *AVPR1A*, and *AVPR1B* genes for each individual locus are presented in Table 1.

### 3.2. Population Differentiation with Respect to AVP, AVPR1A, and AVPR1B Genes

The strongest genetic population differentiation was found in the case of the *AVPR1B* gene. For the GRB to ACB and CHB to ACB populations, the mean Fst for all *AVPR1B* gene variants was 0.321. At the same time, rs28499431, rs33940624, rs28477649, rs3883899, and rs28452187 showed the highest Fst values in all populations under analysis (Table 2). As the frequency of the preferred allele rises rapidly, the classic strong directional selection signal tends to be localized to an unusually long invariable haplotype. According to the results of the iHS analysis, all SNVs of the *AVPR1B* gene were subject to positive selection in the GBR populations (Table 2). A similar phenomenon was observed in the FIN, IBS, and PUR populations, in which almost all SNVs of the *AVPR1B* gene showed a positive selection signature. In contrast, four positively selected SNVs were identified in the SlEast population, and only one SNV was identified in the BEB, CHB, and ACB populations.

Based on the results of nSl analysis, the selection was confirmed for all SNVs of the *AVPR1B* gene (with the exception of rs28588803 in GBR, which showed a positive signal detected by the iHS method). Additional SNVs have been identified in all populations except ACB.

Based on an assessment of iHH12, selection signals in the *AVPR1B* gene were identified only in the GBR population. In other populations, positive selection signals were not detected by the iHH12 method (Table 2).

Variants in the *AVPR1A* gene also showed significant multipopulational divergence relative to the ACB population. Two variants, rs10784339 and rs10747983, have been identified in the SlEast, GBR, IBS, and PUR populations; two variants, rs11829406 and rs11829452, in the SlEast, IBS, PUR, BEB, and CHB populations and in the SlEast, PUR, BEB, and CHB populations, respectively; and the rs10047514 variant in the GBR and FIN populations (Table 3). Selection traits in the *AVPR1A* gene were found only in the GBR population; two variants (rs11829406 and rs11829452) were identified by the iHS and nSL methods, and four variants (rs10747983, rs10784339, rs10877968, rs1565878685) by the nSL method (Table 3).

The analysis of Fst indicators revealed the absence of significant differentiation signals between ACB and other populations in relation to the *AVP* gene (Table 4). At the same time, a selection signal was found in the ACB population in the *AVP* gene (variant rs3787482) (Table 4).

### 3.3. Population Differentiation with Respect to OXT and OXTR Genes

Analysis of SNVs of the *OXTR* gene generally did not reveal differentiation; however, some variants showed rather high Fst values. Specifically, the levels of differentiation between ACB and CHB in relation to rs2324728l, rs237884, rs1042778, and rs237895, Fst were above 0.5, and between ACB and BEB in relation to rs2324728l and rs237884, Fst reached 0.5 (Table 5). In addition, rs59190448 has been identified in all populations except PUR.

The selection signals identified in the OXTR gene were found in all populations except CHB. The largest number of positively selected SNVs (13 SNVs) was found in the PUR population using the iHS method (Table 5). Three SNVs were identified in the SlEast and ACB populations using the iHS method. At the same time, the rs34992398 variant showed positive selection in the PUR, SlEast, and ACB populations. The rs918316 variant was detected in the GBR population using the iHS method. This variant was also present in the IBS population; however, here, it was discovered using the nSl method. Two SNVs were identified in the BEB population based on the nSl approach. Positive selection of the rs237888 variant was detected in the FIN population by the iHS and nSl methods.

Analysis of Fst scores revealed the absence of significant differentiation signals between ACB and other populations in relation to the OXT gene (Table 4). Of note, a selection signal was also found in the OXT gene; the rs111869749 variant was found in the GBR population (Table 4).

## 4. Discussion

The selection of specific genetic structures in human populations occurs under the pressure of various factors. In general, it aims at the fixation of adaptive changes and, in particular, at the consolidation of complex behavioral programs, which conduce the survival of the individual and the viability of an entire population. Genetic studies make a prodigious contribution not only to understanding the evolution of behavioral traits but also to identifying functional gene variants that influence behavior such as caregiving, tribal care, and cooperation, as well as competition, aggression, and stress response.

In our study, we showed that genes of the oxytocin and vasopressin systems are objects of positive selection in the population process. The selection evolves individually in each population and provides a variety of behavioral reactions. The significance of neuropeptides OXT and AVP and their receptors OXTR, AVPR1A, and AVPR1B is determined by the role they play in the regulation of social interaction and stress response, as well as in maintaining water homeostasis and carbohydrate metabolism. Respectively, the selection in the genes of the oxytocin and vasopressin systems can be forced by a combined driving force.

We found that variants of the OXT, OXTR, AVP, AVPR1A, and AVPR1B genes showed positive selection signals in populations belonging to Indo-European ethnic groups (SlEast, GBR, FIN, IBR, BEB), in the Han subpopulation (CHB), and in Puerto Ricans of Puerto Rico (PUR), who are of a mixed ethnic origin. Overall, the genetic differentiation of the scrutinized populations was stable, as evidenced by the high Fst fixation scores for the analyzed genes in all populations. It is noteworthy that the selection of SNVs of the AVPR1B gene was the most pronounced. SNVs of other genes showed selection signs that were characteristic only for selected populations.

The observed high differentiation of populations in relation to the AVPR1B gene confirms the results of the recent study [36], which indicated a positive selection for the AVPR1B gene both in the phylogenetic lineage of human primates and among populations of White Caucasians, African Americans, Yoruba Africans, and East Asians. Taking into account the role of the AVPR1B receptor in insulin secretion [37,38], the presence of significant positive selection for this gene in representatives of GBR, FIN, IBS, and PUR may be related to the peculiarities of the regulatory hormonal mechanisms of glucose utilization in African Americans and European Americans of European descent. The most notable one is the high secretion of insulin in African Americans [38,39,40,41].

The AVPR1B receptor expression has been shown both in peripheral tissues [42] and in the central nervous system [37,43]. AVPR1B receptor is mainly expressed in the pituitary gland in the brain. It mediates the effect of vasopressin on the corticoliberin-stimulated release of adrenocorticotropic hormone (ACTH) there [44] and, therefore, affects the activity of the hypothalamic-pituitary-adrenal (HPA) axis [45]. One of the hypotheses of the etiopathogenesis of stress-associated diseases, which considers activation of neuroinflammation and inhibition of neuroplasticity, is based on the fact of the HPA axis dysregulation and insufficient control over the basal and stimulated cortisol levels in patients [46,47]. Therefore, it is not surprising that SNVs of the AVPR1B gene are increasingly singled out in GWAS studies as candidates for assessing the risk of the development of major depression and other stress-related psychopathologies. In turn, a fixation of peculiarities of the HPA axis activity occurs at a population level. As had been shown, both basal and stress-stimulated cortisol levels were related to ethnic specificity. The flatter diurnal cortisol slopes were found among African Americans and Hispanics in comparison to Caucasians in the US [48]; in addition, stress-stimulated cortisol secretion during public speaking as well as circadian secretion pattern were reduced in African Americans compared to Caucasians [45]. Since the activity of AVPR1B receptors affects the level of ACTH release, differentiation of populations based on the selection of stress responses of different severity can be mediated by the positive selection of AVPR1B gene variants. Indeed, we found that the rs33933482 variant of the AVPR1B gene, which has been shown to be associated with increased cortisol secretion during public speaking stress in Europeans [45], was positively selected in Indo-European and CHB populations. Other positively selected variants of the AVPR1B gene that we found are known to be associated with side effects of vasopressin [49], panic disorders [18,19], aggression in children [20], and autism spectrum disorders (ASD) [21]. Thus, AVPR1B appears to be one of the genes, the variability of which is underlying the recent adaptation of the stress response.

AVPR1A receptor-mediated signaling is deeply involved in the regulation of physiological functions. It is determined by the effects of vasopressin on glycogenolysis in the liver, insulin secretion, contraction of vascular smooth muscle cells, water reabsorption in the renal tubules (antidiuretic activity), and platelet aggregation [50,51]. The importance of the AVPR1A gene in the control of social interaction, altruistic behavior, and pairing in humans has also been documented [36,52,53]. Variants of the AVPR1A gene, which were identified as positively selected in our study, are associated with heroin and general drug addiction (rs10784339) [19], mental and behavioral disorders associated with substance abuse (rs10747983) [54], diabetic status, increased glucose concentration and triglycerides in the blood, and increased body mass index [43,55]. Effects of AVPR1A gene variants related to the function of the nervous system can be interpreted as those which give preference to behaviors that lead to quick results and allow a more liberal risk assessment at any activity [56].

Our analysis of the selection signatures of the AVP, AVPR1A, and AVPR1B genes shows that population differentiation is accompanied by a selection for variants, which supports variability in stress reactivity among individuals and thus determines the diversity of stress-adaptation strategies. The evolutionary strategy of a positive modulation of the stress response, including positive selection in the genes of the vasopressin system, has certain benefits. The intensive response to stress has advantages from a short-term perspective since it supports a quick and balanced response to changing environmental conditions at any physiological level, including the metabolic and immune systems [36]. The directed selection of vasopressin system genes may result from the relatively recent spread of human populations with settlements in strictly different ecosystems. 

Our analysis showed that, in contrast to the vasopressin system, the OXT and OXTR genes are main subjects of a neutral evolution, which occurs due to genetic drift. The relative stability of the OXT and OXTR genes in the population process may be due to the exclusive role of this neuropeptide hormone in childbirth (coordinating contraction of myometrial cells), lactation, and maternal behavior. However, we have shown a high level of selection in the OXTR gene in the PUR population; partially selective signals are also found in other populations but not in CHB. Selected OXTR gene variants have been shown to be associated with changes in sensitivity to exogenous oxytocin and contractility of myometrium, as well as (rs4686302) with preterm birth [57,58,59]. However, a significant interaction between the effects of rs4686302 and obesity and gestational diabetes cannot be overruled. Our analysis showed that the rs4686302 variant was identified only in the PUR population. This population is characterized by a preterm birth rate of 11.6%, which is higher than averages of 8.1% for other countries of Latin America and the Caribbean, but close to the average rate of 11.9% for African populations [60]. It should be noted that the actual phenotypic significance of this variant in the PUR population is difficult to ascertain due to the combination of the multifactorial nature of preterm birth and the presence of a large percentage of the West African ethnic component in the PUR population.

The neuropeptide OXT is also involved in the regulation of behavior. It determines the degree of affiliation in interpersonal interactions by increasing the efficiency and functional significance of neural networks involved in the control of social interactions [61]. We have identified several variants of the OXT and OXTR genes that were under directed selection. Among them, the rs53576 OXT gene variant showed an association with the level of empathy; OXT gene variants rs6133010 and rs53576 were shown to be associated with the level of emotional intelligence in Western Slavs [54]. Carriers of the G/G genotype in the OXTR rs1042778 gene variant are less sensitive to perceived family support received in order to interfere with the effect of low maternal emotional warmth and acceptance during early life [62]. The OXTR gene variant rs6770632 is associated with depressive symptoms [22]. In humans, the genetic variability in the OXTR rs2254298 variant determines the volume of the amygdala, a brain structure involved in the control of fear and anxiety. In turn, the OXTR rs53576 gene variant is associated with an increased level of stress-stimulated cortisol secretion [63]. According to the results of our analysis, other positively selected variants of the OXTR gene are associated with aggressive behavior and depressive states [22,23,63], alcohol abuse and associated aggressive behavior [24,64], severe schizophrenia, and autism spectrum disorders [23,24,65]. Although the selection for SNVs of the OXTR gene was presumably influenced by the same factors that determined the effective differentiation of populations with respect to the AVPR1A and AVPR1B genes, it should be noted that the exceptional importance of the oxytocin system in the physiological regulation of labor set apart the corresponding genes to a conservative pool that had determined the low the degree of populations’ differentiation in this gene.

Table 6 summarizes the publications on the functional significance of AVPR1A, AVPR1B, and OXTR gene SNVs.

## 5. Conclusions

An analysis of the selection signatures of isolated genomic regions of individuals, which was carried out using general population genetic models, allowed us to show positive selection signals in the *AVP*, *AVPR1A*, and *AVPR1B* genes, as well as, to a lesser degree, in the *OXT* and *OXTR* genes. Positive selection in these genes was actualized with the individual scenario in each case of population differentiation. High population differentiation was revealed with respect to genes of the vasopressin system and, most pronouncedly, to the *AVPR1B* gene, with Fst varied from 0.168 to 0.325. The significance of the *OXT* and *OXTR* genes in the control of labor (a complex of endocrine, paracrine, and immunological mechanisms providing parturition) and the maintenance of maternal behavior was, possibly, the factor determined their greater stability in population formation, which was shown in our study. In general, our results indicate that during the formation of populations, there was a positive selection towards variants that determine the diversity of emotional stress reactions. Despite the elevated probability of accumulation of individuals prone to increased stress-reactivity upon realization of the effects of these SVNs, such selection may be an advantage since it supports immediate behavioral and physiological changes, which result from a balanced reaction of the neuronal, metabolic, and immune systems upon acute environmental stimuli.

## Figures and Tables

**Table 1:** Fst estimates between ACB and other global populations. Each population group is represented by 50 individual data.

	GBR	SlEast	FIN	IBS	PUR	BEB	CHB
***AVP***	0.1256	0.1323	0.1322	0.1241	0.0953	0.0958	0.0967
***AVPR1A***	0.1677	0.2072	0.1589	0.1573	0.1807	0.0876	0.1222
***AVPR1B***	0.3163	0.1677	0.1929	0.2149	0.177	0.1951	0.3253
***OXT***	0.0000	0.0000	0.0000	0.0100	0.0118	0.0000	0.0000
***OXTR***	0.0908	0.0858	0.1060	0.076	0.0527	0.1107	0.1503

**Table 2:** Result of selection mapping in the *AVPR1B* gene. Abbreviations: chr—chromosome, pos—position in the given chromosome, rs—reference number of a single nucleotide variant (SNV); ACB—Afro-Caribbeans in Barbados, SlEast—East Slavs, GBR—British from England and Scotland, FIN—Finns in Finland, IBR—Iberian population in Spain, PUR—Puerto Ricans in Puerto Rico, BEB—Bengalis in Bangladesh, CHB—Han Chinese in Beijing; color codes of cells indicate the cases of significance criterion fulfillment; nSl (orange color code)—the number of segregation sites along the length, iHS (blue color code)—the integral index of haplotypes, iHH12 (yellow color code)—the integral index of homozygosity of haplotypes, Fst (green color code)—fixation index; boxes with gray fill indicate cases of exclusion from the analysis (MAF < 0.05).

chr	pos	rs	ACB		SlEast			GBR				FIN			IBS			PUR			BEB			CHB		
			nSl	iHS	nSl	iHS	Fst	nSl	iHS	iHH2	Fst	nSl	iHS	Fst	nSl	iHS	Fst	nSl	iHS	Fst	nSl	iHS	Fst	nSl	iHS	Fst
chr1	206107041	rs28480002																								
chr1	206107356	rs28405931																								
chr1	206108125	rs28570099																								
chr1	206108197	rs28517473																								
chr1	206108533	rs28418396																								
chr1	206109902	rs33935503																								
chr1	206110067	rs33933482																								
chr1	206110072	rs33985287																								
chr1	206110128	rs28415467																								
chr1	206110179	rs28529127																								
chr1	206110345	rs28676508																								
chr1	206110373	rs28632197																								
chr1	206110634	rs28380027																								
chr1	206110702	rs28733981																								
chr1	206110783	rs28607590																								
chr1	206110952	rs28425623																								
chr1	206111170	rs28483632																								
chr1	206111431	rs33971119																								
chr1	206111732	rs28575468																								
chr1	206112039	rs28499431																								
chr1	206112053	rs34792278																								
chr1	206112099	rs34327164																								
chr1	206112821	rs33940624																								
chr1	206113095	rs28477649																								
chr1	206113467	rs28588803																								
chr1	206114223	rs3883899																								
chr1	206115006	rs28452187																								

**Table 3:** Result of selection mapping in the *AVPR1A* gene. Abbreviations: see legend to Table 2.

chr	pos	rs	ACB		SlEast			GBR			FIN			IBS			PUR			BEB			CHB		
			nSl	iHS	nSl	iHS	Fst	nSl	iHS	Fst	nSl	iHS	Fst	nSl	iHS	Fst	nSl	iHS	Fst	nSl	iHS	Fst	nSl	iHS	Fst
chr12	63144342	rs10047514																							
chr12	63144493	rs11829406																							
chr12	63144669	rs11829452																							
chr12	63144678	rs10747983																							
chr12	63144866	rs10784339																							
chr12	63149405	rs10877968																							
chr12	63149775	rs1565878685																							

**Table 4:** The result of selection mapping in the *AVP* and *OXT* genes. Abbreviations: see legend to Table 2.

gene/chr	pos	rs	ACB		SlEast			GBR			FIN			IBS			PUR			BEB			CHB		
			nSl	iHS	nSl	iHS	Fst	nSl	iHS	Fst	nSl	iHS	Fst	nSl	iHS	Fst	nSl	iHS	Fst	nSl	iHS	Fst	nSl	iHS	Fst
*AVP*/chr20	3083464	rs3787482																							
*OXT*/chr20	3071940	rs111869749																							

**Table 5:** The result of selection mapping in the *OXTR* gene. Abbreviations: see legend to Table 2.

chr	pos	rs	ACB		SlEast			GBR			FIN			IBS			PUR			BEB			CHB		
			nSl	iHS	nSl	iHS	Fst	nSl	iHS	Fst	nSl	iHS	Fst	nSl	iHS	Fst	nSl	iHS	Fst	nSl	iHS	Fst	nSl	iHS	Fst
chr3	8751042	rs2324728																							
chr3	8751160	rs4493422																							
chr3	8751899	rs237884																							
chr3	8752038	*rs6770632*																							
chr3	8752859	*rs1042778*																							
chr3	8755409	rs237888																							
chr3	8756495	rs918316																							
chr3	8760793	rs57329700																							
chr3	8761165	rs11131149																							
chr3	8761315	rs59190448																							
chr3	8762109	rs34992398																							
chr3	8762685	*rs53576*																							
chr3	8763680	rs35498753																							
chr3	8764054	rs7652281																							
chr3	8764628	rs11711703																							
chr3	8764738	rs73132848																							
chr3	8765737	rs237895																							
chr3	8766123	rs61183828																							
chr3	8766375	rs6767512																							
chr3	8766599	rs237897																							
chr3	8766747	rs34880121																							
chr3	8767536	rs4686302																							
chr3	8768322	rs237911																							
chr3	8768722	rs4564970																							
chr3	8768834	rs35413809																							
chr3	8768922	rs73132856																							
chr3	8769210	rs2301261																							
chr3	8769543	rs2301260																							

**Table 6:** Evidence for the functional significance of SNVs of genes of the oxytocin and vasopressin system. Abbreviations: rs, reference number of a single nucleotide variant (SNV); ACB—Afro–Caribbeans in Barbados, SlEast—Eastern Slavs, GBR—British from England and Scotland, FIN—Finns in Finland, IBR—Iberian population in Spain, PUR—Puerto Ricans in Puerto Rico, BEB—Bengalis in Bangladesh, CHB—Han Chinese in Beijing; nSl is the number of segregation sites along the length, iHS is the integrated index of haplotypes, iHH12 is the integrated index of homozygosity of haplotypes, and Fst is the index of fixation.

	SNVs of a Positive Selection (Original Data)		Proved Functional Associations (Literature Data)	
	Gene/rs	Method/Population	Phenotype/Endophenotype	Reference
1	*AVPR1A* rs10747983	Fst/ACB & SlEast Fst/ACB & GBR Fst/ACB & IBS Fst/ACB & PUR nSl/GBR	Diabetic status, elevated blood glucose and triglycerides, body mass index (BMI)	Enhörning et al., 2008 [43]
2	*AVPR1A* rs10784339	Fst (ACB& SlEast) Fst (ACB& GBR) Fst (ACB& IBS) Fst (ACB& PUR) nSl GBR	Heroin addiction, drug abuse.	Levran et al., 2014 [66]
			Drug abuse	Maher et al., 2011 [55]
			Diabetic status, elevated blood glucose and triglycerides, BMI	Enhörning et al., 2008 [43]
3	*AVPR1B* rs28632197	iHS—ACB iHH12—GBR nSl—FIN nSl, iHS—IBS	Autism spectrum disorders (ASD)	Francis et al., 2016 [67]
			Panic disorders	Kreek et al., 2011 [19]
			Panic disorders	Keck et al., 2008 [20]
4	*AVPR1B* rs28418396	nSl, iHS, iHH12—GBR nSl, iHS—FIN nSl, iHS—IBS nSl, iHS—PUR nSl—BEB, nSl—HAN	Significant side effects of therapeutic doses of vasopressin and norepinephrine	Anantasit et al., 2014 [18]
5	*AVPR1B* rs33933482	nSl, iHS, iHH12—GBR Fst ACB & GBR nSl, iHS—FIN nSl, iHS—IBS nSl, iHS—PUR Fst ACB & HAN	Psychosocial stress test (public speaking)-evoked plasma cortisol levels	van West et al., 2010 [45]
6	*AVPR1B* rs28676508	nSl, iHS, iHH12—GBR Fst ACB & GBR nSl, iHS—FIN nSl, iHS—IBS nSl, iHS—PUR Fst ACB & HAN	High child aggression.	Zai et al., 2012 [21]
7	*OXTR* rs237884	Fst ACB& HAN Fst ACB& BEB nSl BEB	Symptom severity and treatment outcomes in subjects with schizophrenia	Souza et al., 2010 [24]
			ASD	Wermter et al., 2010 [65]
			Changes in social functioning in ASD	Harrison et al., 2015 [68]
8	*OXTR* rs6770632	Fst ACB& HAN Fst ACB& BEB nSl BEB	Alcohol abuse in adolescents and young adults	Kim et al., 2018 [64]
			Child aggression	Malik et al., 2012 [23]
			Extreme, persistent, and pervasive aggressive behaviors in females and males	Malik et al., 2012 [23]
9	*OXTR* rs1042778	Fst ACB& HAN	High scores of depressive symptoms	Keijser et al., 2021 [22]
			Aggressive behavior in boys	Malik et al., 2012 [23]
10	*OXTR* rs237888	nSl FIN	Increased oxytocin sensitivity of human myometrial cells *in vitro*	Füeg et al., 2019 [57]
			The value of the effective dose of oxytocin and the outcome of childbirth	Grotegut et al., 2017 [69]
11	*OXTR* rs59190448	Fst ACB& SlEast Fst ACB& GBR Fst ACB& FIN Fst ACB& IBS Fst ACB& BEB Fst ACB& HAN	Positive selection	Schaschl et al., 2015 [70]
12	*OXTR* rs61183828	iHS/ACB	Liver fibrosis in patients with human immunodeficiency virus/hepatitis C virus coinfection	Ulveling et al., 2016 [71]
13	*OXTR* rs4686302	iHS/PUR	Increased oxytocin sensitivity of human myometrial cells *in vitro*	Füeg et al., 2019 [57]
			Deficit in social cognition in individuals with Attention Deficit and Hyperactivity Disorder (ADHD)	Kalyoncu et al., 2019 [72]
			Alcohol abuse in adolescents and young adults	Kim et al., 2018 [64]
			Preterm birth	Kim et al., 2013 [58]
			The need for high doses of oxytocin in parturients	Reinl et al., 2017 [59]
14	*OXTR* rs4564970	iHS/PUR	Antisocial behavior of teenage boys	Hovey et al., 2015 [73]
			High exogenous oxytocin sensitivity	Chen et al., 2015 [74]
			Aggressive behavior under alcohol intoxication	Johansson et al., 2012 [75]
			Panic disorders	Kreek et al., 2011 [19]
			Panic disorders	Keck et al., 2008 [20]

## Data Availability

Data are available on demand.

## Supplementary Materials

The following supporting information can be downloaded at: https://www.mdpi.com/article/10.3390/genes14112053/s1, Figure S1: Sample stratification was based on the principal component analysis (PCA) of the first two components, which was performed with the plink software and visualized with the ggplot2 (R package); Figure S2: Ancestry analysis by admixture. The number of K ancestral clusters was varied from 2 to 8. Individuals were grouped according to the subpopulation assignments made by PCA (Figure S1).
