# Supplementary material for: Genomic Signatures of Positive Selection in Human Populations of the OXT, OXTR, AVP, AVPR1A and AVR1B Gene Variants Related to the Regulation of Psychoemotional Response
Source: Genes (Basel). 2023 Nov 8;14(11):2053. doi: 10.3390/genes14112053 (PMC10670988; doi:10.3390/genes14112053)
Supplement: Supplementary file 1 [file genes-14-02053-s001.zip › genes-2665007-supplementary.pdf]

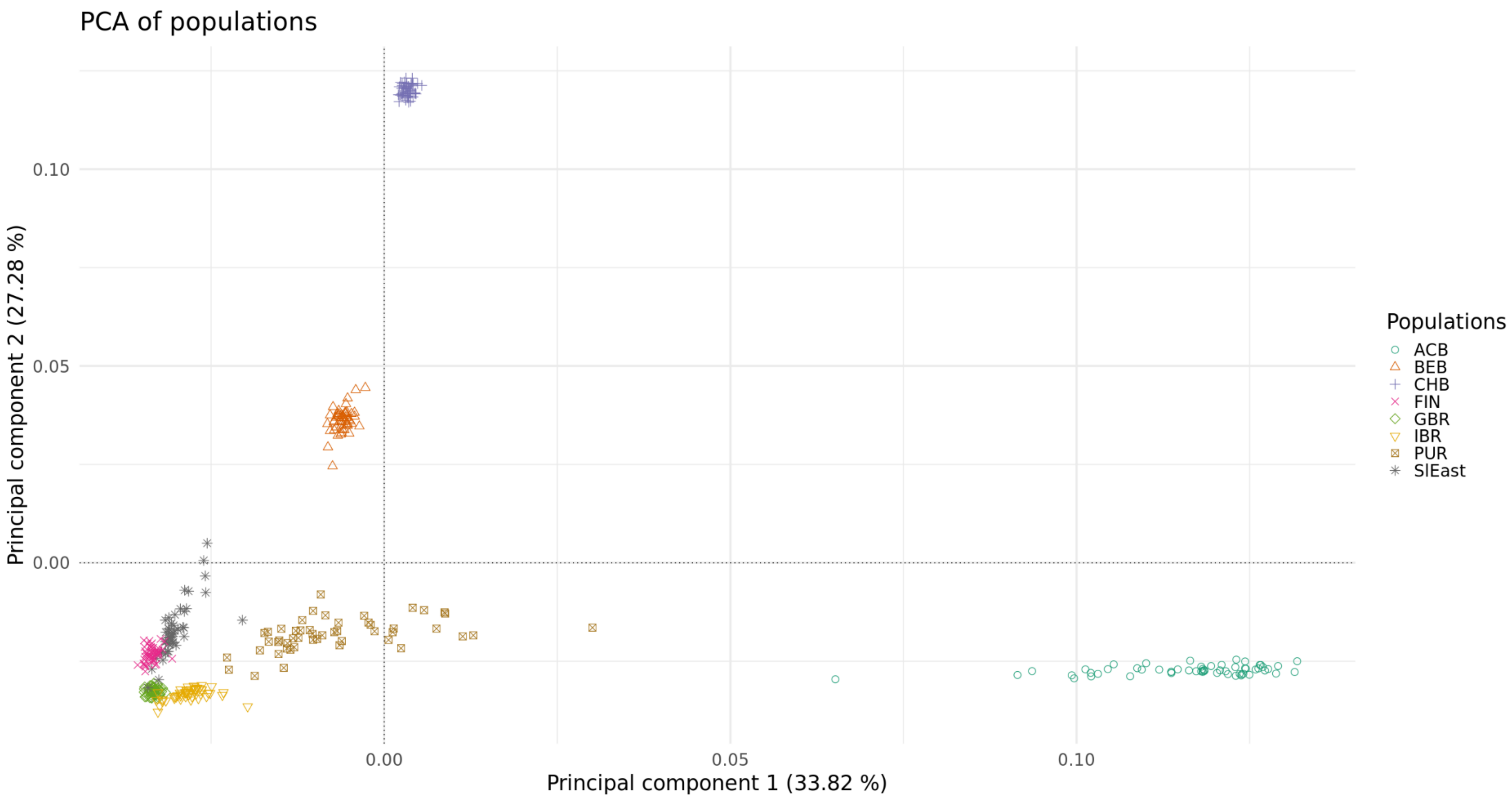

Figure 1S. Sample stratification was based on the principal component analysis (PCA) of the first two components, which was performed with the plink software and visualized with the ggplot2 (R package). Abbreviations: Afro-Caribbeans in Barbados, SlEast - East Slavs, GBR - British from England and Scotland, FIN - Finns in Finland, IBR - Iberian population in Spain, PUR - Puerto Ricans in Puerto Rico, BEB - Bengalis in Bangladesh, CHB - Han Chinese in Beijing.

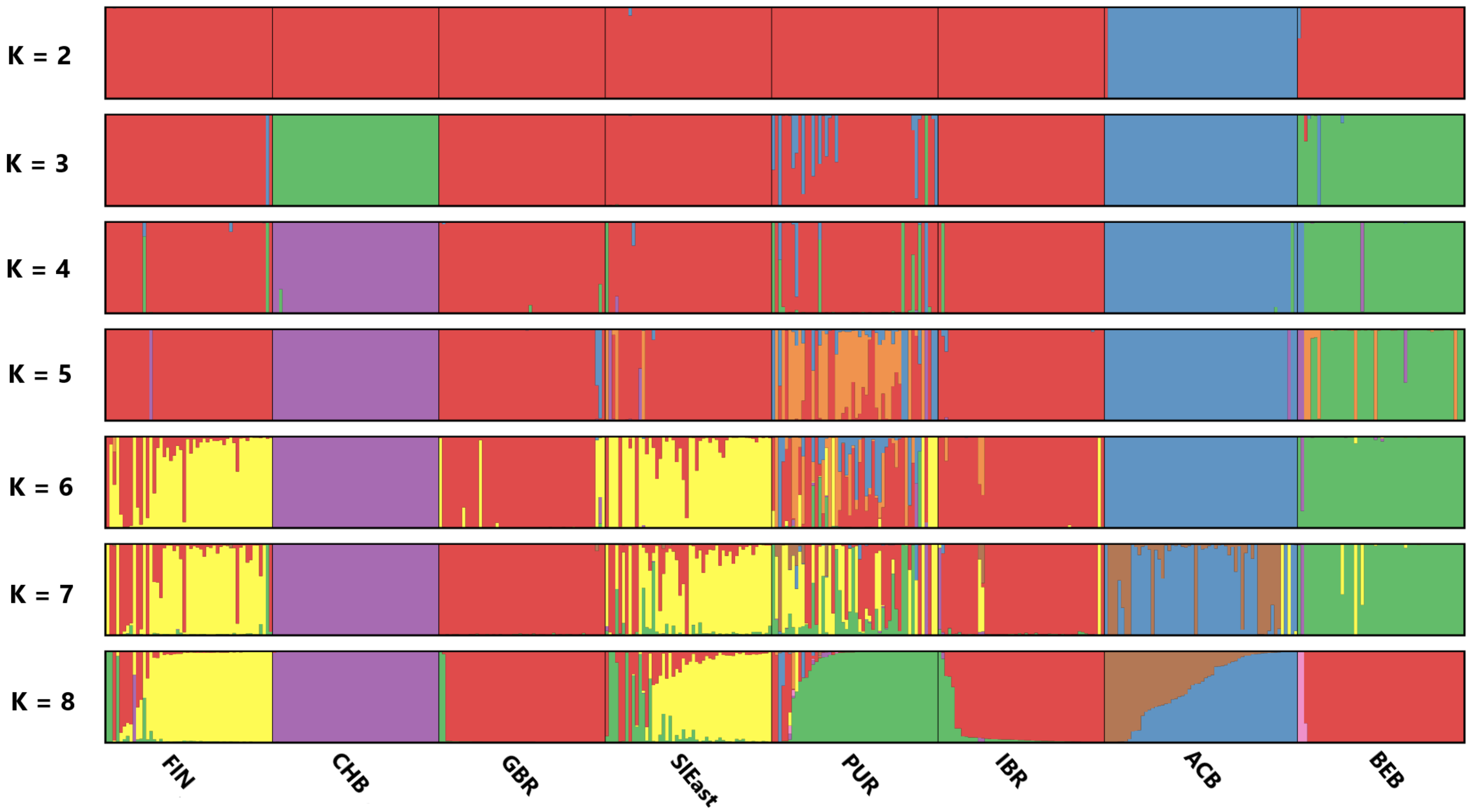

Figure 2S. Ancestry analysis by *admixture*. The number of K ancestral clusters was varied from 2 to 8. Individuals were grouped according to the subpopulation assignments made by PCA (Figure 1S). Abbreviations: see Figure 1S.
